# Immunoprevention of non-viral cancers: challenges and strategies for early intervention

**DOI:** 10.1186/s12935-025-03817-8

**Published:** 2025-05-28

**Authors:** Kajal Biswas, Lillian S. Kuo, Robert H. Shoemaker, Altaf Mohammed

**Affiliations:** 1 Chemopreventive Agent Development Research Group, Division of Cancer Prevention, National Cancer Institute, Rockville, MD USA; 2 Cancer Immunology, Hematology and Etiology Branch, Division of Cancer Biology, National Cancer Institute, Rockville, MD USA; 3 Chemopreventive Agent Development Research Group, Division of Cancer Prevention, National Cancer Institute, 9609 Medical Center Drive, Room no: 4E454, Rockville, MD 20850 USA; 4 Chemopreventive Agent Development Research Group, Division of Cancer Prevention, National Cancer Institute, 9609 Medical Center Drive, Room no: 5E602, Rockville, MD 20850 USA

**Keywords:** Immunoprevention, Cancer prevention, Neoantigens, Cancer vaccine, Cancer interception

## Abstract

While the effects of cancer vaccines have been extensively studied in the therapeutic setting, research has been more limited in the areas of cancer prevention and interception. Although cancer prevention by vaccines has been possible for viral-mediated cancers, such as cervical cancers and hepatocellular carcinoma, preventing non-viral cancers by immunopreventive vaccines is challenging. Many tumors at late stages are less responsive to treatments, including immunotherapies and vaccines, in part due to an immunosuppressive microenvironment. Shifting the strategy to intervention at early stages of cancer development and progression and focusing on high-risk cohorts with defined molecular targets offers a pathway for improved vaccine efficacy. Current research on the role of immune mechanisms during tumor initiation and progression is rapidly evolving and recent emerging preclinical immunoprevention studies have shown that vaccines can induce host immune response and effectively control tumor onset and progression. In this review, we address important considerations and challenges regarding the development of cancer immunoprevention for non-viral cancers. We also discuss significant, innovative, and impactful preclinical and clinical immunoprevention studies in various cancers. This includes neoantigen discovery, the use and optimization of immunomodulating agents either alone or in combination with vaccines, and strategies for optimizing vaccines. We conclude by discussing prospects for immunoprevention research and potential opportunities to advance the field in the future.

**Supplementary Information:**

The online version contains supplementary material available at 10.1186/s12935-025-03817-8.

## Introduction

**Fig. 1:**
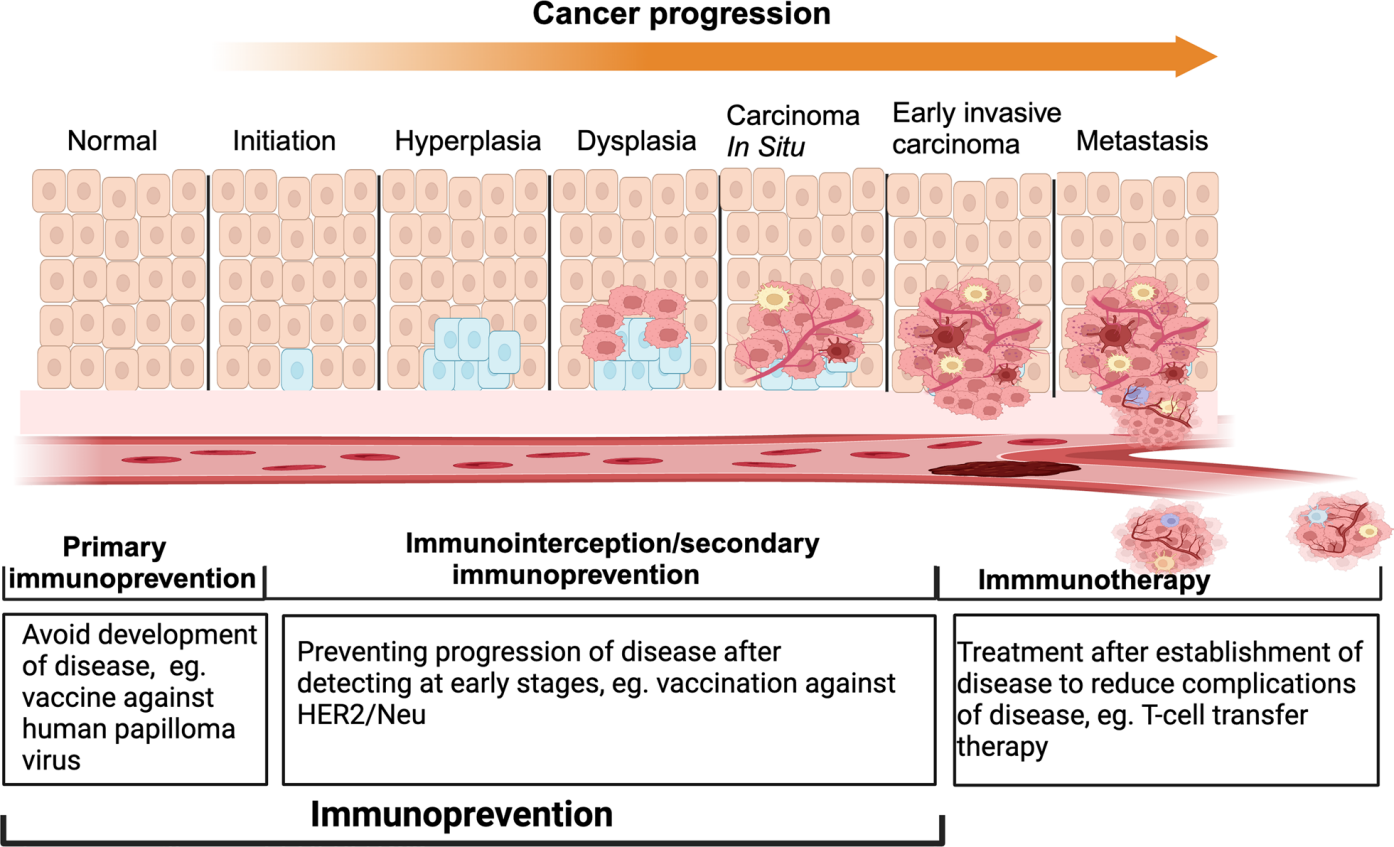
Stages of tumor development and immune system based interventions for initiation and progression. Immunoprevention strategies are efficacious during the cancer initiation and early progression. Primary immunoprevention is focused to reduce the chance of cancer initiation whereas, secondary immunoprevention strategies help to inhibit the progression of early cancer or pre-cancer lesions. After the disease is established, immune system based interventions are referred as immunotherapy and used to reduce the complications associated with the disease

Cancer initiation and progression is dependent on genetic and environmental risk factors and they are tightly interconnected with the immune system [1]. In general, preventing the development of cancer involves taking action to lower the risk of developing cancerous cells that can be achieved by avoiding exposure to known cancer-causing substances, maintaining healthy lifestyles, and using prophylactic vaccines. These risk-reducing measures are considered *primary prevention* as they aim to prevent the initiation or development of malignancy. Along with avoidance of cancer risk factors, cancer prevention strategies also include active interventions that lead to the diagnosis and control of precancerous lesions at the earliest stage possible (Fig. 1). Cancer interception refers to disruption of the oncogenic process during the precursor or precancer state or stage before the development of invasive cancer, which is considered *secondary prevention* (Fig. 1). Cancer immunoprevention involves using immunological means such as vaccines or immunomodulatory agents to prevent cancer initiation (primary prevention) or invasive cancer onset (secondary prevention or interception) [2] (Fig. 1). In this review, we collectively refer to both cancer prevention and interception measures using immunologic approaches as immunoprevention.

**Fig. 2:**
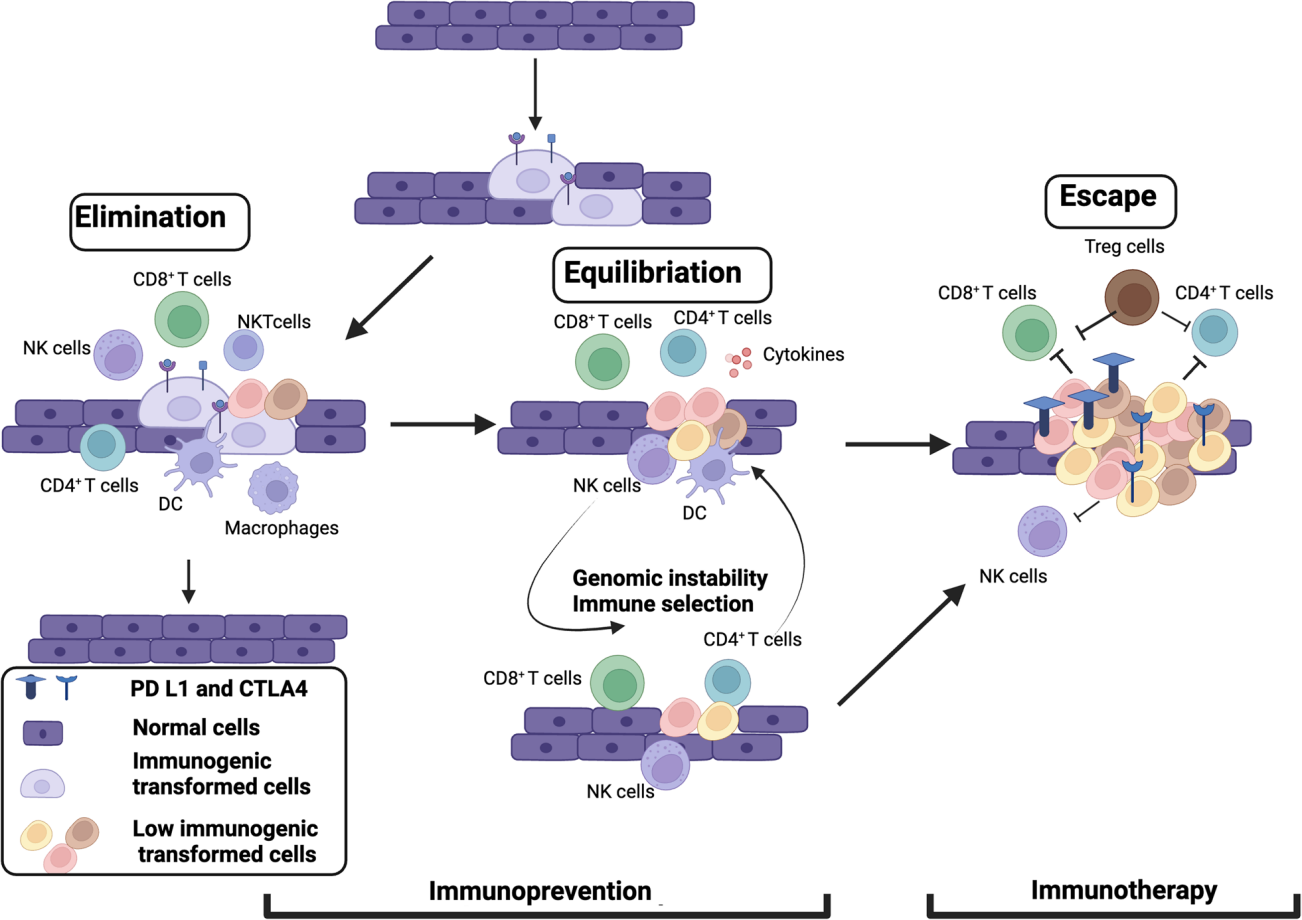
Cancer immunoediting phases and timing for immunoprevention and immunotherapy. At elimination phase, transformed tumor cells are recognized and removed before clinical detection by synergistic action of the innate and adaptive immune systems. A balance is established between the tumor cells and the immune system at equilibrium phase and premalignant lesion can arise. Prevention strategies using immune system should be administered during this stage to eliminate tumor cells. The last phase is the escape phase when immune system is ineffective to control tumor cell growth and proliferation and immunosuppressive microenvironment is established in combination of tumor and stromal cells. Immunotherapy is targeted to activate immune system or to overcome immunosuppressive environment to clear the tumor cells

The key difference between immunoprevention and immunotherapy lies in the timing of events and the expected outcome (Figs. 1 and 2). Immunotherapy is used when the disease is established and diagnosed and given to clear the malignant invasive or metastatic tumors. Immunotherapeutic approaches include augmenting anti-tumor immune responses by vaccination and/or administration of immune stimulatory cytokines as well as the use of checkpoint inhibitors. Immunoprevention approaches by vaccines or immunomodulatory agents on the other hand prevent lesion formation, or detect and eradicate premalignant lesion or intercept the progression of lesions or precancers to invasive cancers (Fig. 2). Immunoprevention approaches could be best applied for cancer interception for individuals who have developed pre-cancerous lesions, for example, gastrointestinal polyps, prostatic intraepithelial neoplasia (PIN), or intraductal papillary mucinous neoplasms (IPMNs) that could develop into colorectal tumors, prostate cancer or pancreatic cancers respectively. Moreover, immunosuppression, a major issue in treating established invasive or metastatic cancers through immunotherapy, is less induced in early neoplasms. Therefore, the clinical impact of immunoprevention could be greater than immunotherapy.

Effective vaccines are available for the cancers caused by viruses, such as vaccines against human papillomavirus for preventing cervical cancer [3] and vaccines against hepatitis B that reduce the incidence of liver carcinoma[4–8]. However, as the majority of cancers are non-viral mediated, developing immunoprevention strategies without significant toxicities and providing long-term protection is challenging. Moreover, identifying immunoprevention-specific targeting antigens poses another challenge for non-viral cancers due to the large variability of tumor antigens, and this research is still at the pre-clinical stage of development [2]. At this point, it is highly unlikely to have a generic immunoprevention vaccine strategy that can target all non-viral tumors in the general healthy population. For non-viral cancers, immunoprevention will be beneficial for human populations who are subject to a predictable risk of developing certain tumors like carriers of pathogenic mutations in tumor suppressor genes, environmental/occupational exposure to carcinogens etc.

Several preclinical studies have shown that early induction of host immune responses can delay tumor onset and progression in animal models of different cancers [9–12]. In this review, we will discuss important considerations for cancer immunoprevention and summarize current preclinical and clinical studies for immune based non-viral cancer prevention.

## Role of the immune system from tumor initiation to progression

For the past two decades, considerable efforts have been directed towards understanding the impact of immune cells at various stages of tumor development: early neoplastic development, clinically detected tumors, and metastatic progression. Whether or not inflammation is a cause or consequence of cancer, it is clear that the presence of innate and adaptive immune cells is a feature of cancer progression. Genomic instability of cancer cells results in the accumulation of mutations and generation of tumor antigens. Immunogenic cancer cells are eliminated during the early phase of tumor initiation by both innate and adaptive immune systems. The cytotoxic T cells like CD8^+^ T cells and NK (Natural Killer) cells play significant roles during the elimination process. NK cells are activated by binding of NK cell receptors to the ligands produced by tumor cells due to DNA damage or oncogenic pathway activation. Activated NK cells produce cytotoxic molecules, including perforin and granzyme that can eliminate tumor cells and activate apoptotic pathways in tumor cells by producing TNFα [13]. CD8^+^ T cells are components of the adaptive immune response and are activated upon priming by antigen presenting cells (APC) to differentiate into cytotoxic T lymphocytes (CTLs) [14]. The CTLs then destroy the target cells by releasing perforin and granzyme molecules. Cytokines released by CD4^+^ T helper 1 cells such as IL2, TNFα, IFNγ, promote anti-tumoral activity of NK cells, T-cell priming, activation and CTL cytotoxicity [13, 15].

Less immunogenic tumor cells escape the elimination phase and proceed to the equilibration phase of immunoediting where immunologic responses check their outgrowth [16]. Clinically apparent tumor appears when the tumor cells escape immune recognition. Tumor cells may recruit a variety of immune cells to establish an immunosuppressive environment.

Mechanisms for immune escape of tumor cells can be classified into three groups: (1) avoidance of recognition by immune cells, (2) increased mutation in pro-survival genes, and (3) resistance to immune response by promoting the establishment of an immunosuppressive microenvironment. There are number of ways for immune evasion. One example is to avoid recognition by APCs, and for that, tumor cells downregulate the expression of MHC I molecules or strong tumor antigens that are recognized by immune cells or inhibit costimulatory signals to activate APCs [15, 17]. The CTLs thus fail to recognize the antigens on tumor cells leading to enhanced tumor growth. Mutations in genes involved in IFNγ signaling in tumor cells may result in a defect in antigen presentation and diminish T-cell recognition. Tumor cells promote the immunosuppressive environment by variety of mechanisms including upregulating immune checkpoint molecules (e.g.: PD L1), suppressing DC activation by activating the WNT β catenin pathway and by recruiting T regulatory cells and myeloid derived suppressor cells [18]. The presence of immunosuppressive cells has been seen in early premalignant lesions as well [19]. A schematic presentation of elimination, equilibration and escape phases of tumor cells with the potential for immunoprevention strategy is illustrated in Fig. 2 [20, 21].

## Antigen selection for immunoprevention of cancer

An important step for immunoprevention is the choice of antigens that will be targeted. Selection of antigens that elicit immune response with low central tolerance is still an active area of investigation for cancer immunoprevention.

For cancers that are not developed in association with infectious agents, the best target antigen choices are oncoantigens or the molecules that have been involved in the carcinogenic processes. There are two major classes of tumor antigens: tumor associated antigens (TAA) that have elevated levels in tumor cells but also express in healthy cells, and the tumor specific antigens (TSA) that are found only on cancer cells. Genomic instability of tumor cells generates various mutations in coding and noncoding regions of different genes. Amino acid sequence changes due to mutation in the coding regions can generate proteins specific to the tumor cells. Neoantigens or tumor specific antigens that are generated during pre-malignant to malignant conversion of cells or expressed due to exposure to chemical carcinogens are ideal targets for T cells to recognize and induce strong anti-tumor immune response [22–24]. We will discuss below different antigens that have been considered for cancer immunoprevention.

### Tumor associated antigens (TAA)

Tumor associated antigens (TAAs) are host proteins that are significantly over-expressed in tumor cells [22]. To develop a prophylactic vaccine, it is important to identify TAA that expresses in pre-malignant lesions. SOX2 is expressed in embryonic stem cells and a regulator of stemness and pluripotency. In patients with monoclonal gammopathy of undetermined significance (MGUS), a premalignant form of multiple myeloma (MM), the presence of anti-SOX2 T cells and humoral responses correlate with reduced risk of MM progression [23]. This suggests that boosting anti-SOX2 immunity by preventive vaccine can benefit MGUS cohorts.

Another well studied TAA is human epidermal growth factor receptor-2 (HER2). HER2 expression is associated with progression to invasive ductal carcinoma (IDC) of breast from ductal carcinoma in situ (DCIS), an early preinvasive breast neoplasm. HER2-based vaccination can induce long term immune response and eliminates HER2 positive cells from DCIS lesions [24].

A type I transmembrane glycoprotein, mucin 1 (MUC1) is overexpressed in hypoglycosylated form in over 80% of human cancers and is involved in the tumor immune evasion mechanism [25]. A MUC1-based vaccine elicited high IgG antibodies and long-lasting immunogenic memories in 43.6% patients with pre-malignant lesions of colon cancer [26]. Clinical trials are underway using MUC1-based vaccine to prevent lung cancer in current and former smokers and in preventing recurrence of colon polyps in patients with advanced adenomas [25].

Some other TAAs that are also over expressed in pre-neoplastic lesions are cyclin B1, cancer-testis antigens such as MAGEA antigens, sarcoma antigen 1 (SAGE1), cancer-testis antigen 47A (CT47A), and nuclear RNA export factor 2 (NFX2) (Table 1) [27, 28]. Some of these TAAs showed transient abnormal expression due to acute inflammation associated with infections. Women who experienced acute infections early in their life has been shown to generate antibodies against hypoglycosylated MUC1 and have reduced risk of developing ovarian cancer [29–31]. Childhood diseases like chicken-pox or pertussis and repeated influenza infections throughout life have been shown to be associated with reduced risk of developing breast, colorectal, stomach or ovarian cancer [32]. These findings led to a new hypothesis that reducing risk of cancer is associated with effective immune surveillance directed against infection associated transiently expressed self-antigens that later get expressed in tumor cells. Further studies are needed to find these disease associated antigens that later express as TAAs in primary tumor tissues.

**Table 1:** List of some tumor associated antigens that are potential targets for vaccine development

Antigens	Cancer associated	Expression	References
SOX2 (Sex determining region Y-Box transcription factor 2)	Multiple myeloma	Pre cancer and cancer cells	[23]
HER2/Neu (Human epidermal growth factor receptor 2)	Breast cancer	Overexpression in cancer cells	[119]
MUC1 (Mucin 1)	Breast cancer	Abnormal glycosylation and overexpression in cancer cells	[120]
Tyrosinase	Melanoma, leukemia	Expressed in melanocyte only with overexpression in cancer cells	[121]
MAGE-1 (Melanoma antigen 1)	Melanoma	Expressed in melanocyte with overexpression in cancer cells	[122]
SAGE-1 (Sarcoma antigen 1)	Head and neck squamous cell carcinoma, esophageal squamous cell carcinoma	Cancer cells	[27, 123]
Cyclin B1	Prostate, Head and neck, lung	Overexpression in cancer cells	[124, 125]
CT47A (Cancer testis antigen 47A)	Esophageal cancer	Pre cancer lesion and cancer cells	[27]
hTERT (Human Telomerase Reverse Transcriptase)	Melanoma, Leukemia	Overexpression in cancer cells	[126]
CA-125 (Cancer antigen 125)	Ovarian cancer	Overexpression in cancer cells	[127]
gp100 (Glycoprotein 100)	Melanoma	Expressed in melanocyte with overexpression in cancer cells	[128]
Alpha-fetoprotein	Hepatic cancer	Higher expression in cancer cells	[129]
CEA (Carcinoembryonic antigen)	Colon cancer, lung cancer	Higher expression in tumor cells	[130]
EGFR (Epidermal growth factor receptor)	Pancreatic, colorectal, breast, lung	Higher expression in tumor cells	[131]
Prostein	Prostate cancer	Prostate specific expression	[132]

Moreover, central tolerance of reactive T-cells against self-antigens remains an important consideration while using TAAs as vaccine targets. Immunomodulatory approaches like checkpoint inhibitors and/or targeting regulatory networks that restrain tumor immune responses may be considered in combination with vaccines against TAA to induce robust immune responses [33].

### Neoantigens

Neoantigens or tumor specific antigens (TSA) are peptides that are produced in tumor cells due to a variety of genetic alterations and recognized by neoantigen specific T-cell receptors (TCRs) in the context of major histocompatibility complexes (MHCs). These neoantigens are generally absent in normal human cells. The mutation derived neoantigens induce the immune system to target and eliminate the neoantigen expressing cancer cells.

To develop a prophylactic vaccine, it is important to select shared neoantigens that are present in multiple individuals’ tumors. The current surge of tumor genome sequencing and development of machine learning algorithm to select immunogenic neoepitopes have successfully selected neoantigens for some cancers [34, 35]. There are some neoantigens that appear in every tumor that originates from certain mutations. For instance, the majority of pancreatic cancers bear mutation in KRAS protein that can give rise to a predictable neoantigen. In a genetically engineered pancreatic cancer mouse model (*Kras-p53-Cre*), immunization with a vector encoding *Kras*^*G12D*^ mutation in combination with cyclophosphamide Treg depletion significantly slowed the progression of early PanINs compared to control mice [36]. Recently, a phase I clinical trial was initiated to test the safety and immune response of vaccination using pooled mutant-KRAS neoantigens in high-risk group (NCT05013216). A predictable set of neoantigens are generated in tumors of Lynch syndrome (LS) patients who carry mutations in DNA mismatch repair proteins. The defective DNA repair system generates “frameshift” peptides (FSPs). A phaseI/IIa clinical trial with several of these antigens showed safety and immunogenicity in patients with microsatellite instable (MSI) colorectal cancer [37]. Vaccination using four FSP neoantigens in a Lynch syndrome mouse model reduced intestinal tumor burden and prolonged overall survival [12]. Considering the heterogeneity among cancer cells and heterogenous immune responses to neoantigens among population, a phase Ib/II clinical trial with vaccination using 209 Lynch tumor FSP neoantigens in patients who carry LS mutations (NCT05078866) is underway. Tumor specificity, ability to boost immune mechanisms and the development of techniques for identifying and selecting neoepitopes point towards a bright future for neoantigen based prophylactic vaccines.

### Immunogenic neoantigen selection and validation

**Fig. 3:**
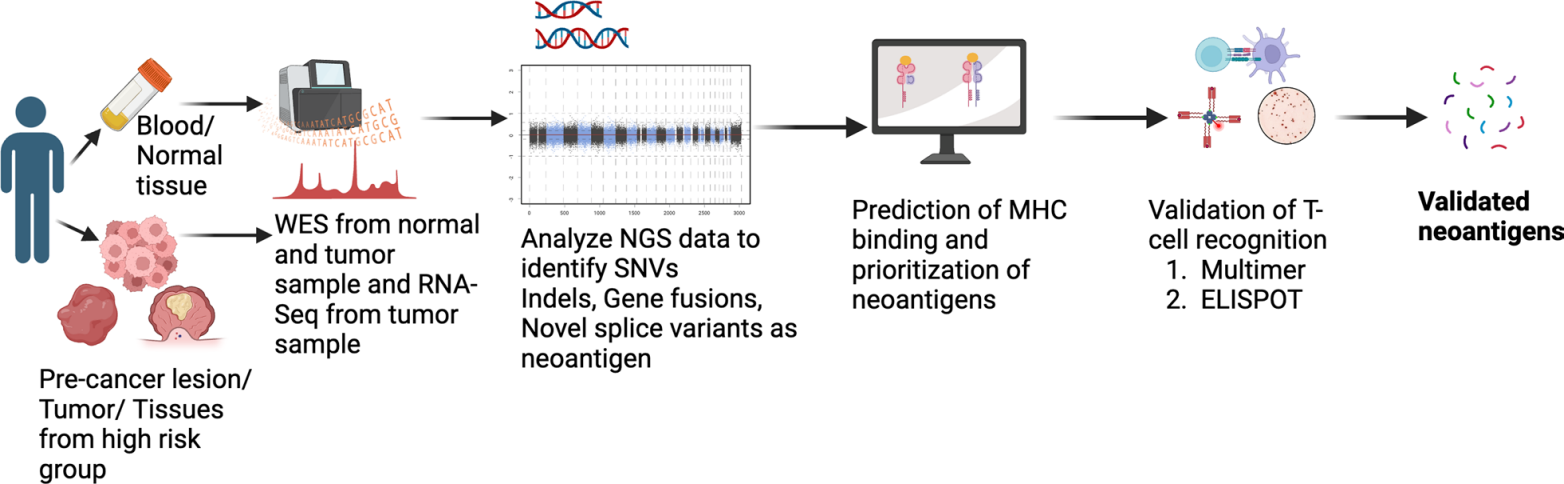
Workflow for a neoantigen prediction and validation pipeline. Whole-exome sequence data (either from precancers, lesions, tumor or tissues from high-risk group) and RNA sequencing data are analyzed for (1) *HLA* genotypes, (2) somatic mutations, and (3) the expression levels of mutated genes and possible neoantigens are further estimated *in silico* for their affinities to major histocompatibility complex (MHC) molecules. Predicted neoantigens can then be validated for their ability to activate T-cells using assays like ELISPOT or multimer assay

Neoantigen prediction and prioritization are required for successful identification of immunogenic neoantigens. A schematic for neoantigen prediction and validation is summarized in Fig. 3. Neoantigen prediction involves sample acquisition (either from precancers, lesions, tumor or tissues from high-risk group), high quality sequencing data, identifying the mutations present in the sample and prediction of neoantigens resulting from these mutations. Prediction of neoantigens follows prioritization based on the prediction of their potential to elicit CD8^+^ or CD4^+^ T cell response. For immunogenicity prediction of neoantigens, a variety of computational tools have been developed. Those tools are used to predict each step like MHC class I restricted neoantigen prioritization, expression of neoantigen, proteasomal cleavage, binding potential to MHC class I or class II molecule, stability of neoantigen, MHC interaction and potential to be recognized by T cell receptors (TCR). Various models have been developed to integrate predictions from these individual tools into an overall score of immunogenicity prediction, as reviewed elsewhere [38–40]. Among the different integrated models for calculating neoantigen immunogenicity score, the one commonality for all models is the inclusion of MHC binding affinity. Two models (TESLA and NeoScore) selected the same three characteristics: MHC class I binding affinity, MHC class I binding stability and mRNA expression with a difference that NeoScore provides a continuous score while TESLA provides a series of thresholds over the selected characteristics [39, 41]. The selection of different characteristics to derive immunogenicity score by these models reflect their differences on the underlying training datasets [38]. Further research is needed to select the best set of characteristics to derive neoantigen immunogenicity score. The success of neoantigen prediction has been biased towards MHC class I-specific neoantigens due to difficulties to find predictable binding motif for highly diverse MHC class II molecules. This highlights the need for new MHC class II prediction algorithm [42].

Selected neoantigens can then be validated in different ways. The common validation assays are mass spectrometry, MHC multimer staining, ELISA, ELISpot or intracellular cytokine staining. These techniques measure different features of neoantigens: mass spectrometry validates the binding of neoantigens to MHC molecules [43], MHC multimer staining validates TCR recognition and expansion of neoantigen-specific T cells[44] and ELISA, ELISpot or cytokine staining validates activation of T cells [38]. Combining two or more techniques may provide better confirmation of neoantigen immunogenicity. A few validated datasets focused on SNV and small indel derived neoantigens for melanoma, lung or ovarian cancers are available now and are reviewed by Borden et al. [38]. Expansion of the validated neoantigen datasets for expanded sets of mutations and for various cancers are needed in the future to enhance the clinical application of neoantigen based cancer immunoprevention. Neoantigen prediction, prioritization and validation for cancer vaccine development are rapidly expanding and a combination of datasets along with improvement of computational modelling will help to build clinically relevant models in the future.

## Selection of high-risk populations for cancer immunoprevention

In cancer immunoprevention, a major obstacle is that every non-viral cancer is unique, and no universal vaccine can be used for all types of cancers. It is difficult to predict among the general population the occurrence of a particular cancer unless the population has certain genetic mutations or a predisposition factor of a specific cancer. A generalized cancer vaccine is currently an impossible task and the target for cancer immunoprevention is thus high-risk populations prone to developing specific cancers. Here we describe some examples of high-risk population and the possible ways to develop effective cancer prevention vaccines for these individuals.

Individuals who inherit mutations in mismatch repair (MMR) genes develop Lynch syndrome (LS) or constitutional MMR deficiency syndrome (CMMRD) and are highly susceptible to development colorectal or other types of cancers [45]. The cancers developed in those population display somatic hypermutation phenotype due to MMR defects. Microsatellite repeats loci present in coding regions accumulate frameshift mutations due to inefficient mismatch repair during replication of DNA and generate frameshift mutation derived peptides (FSP) that are highly immunogenic in nature. The resulting neoantigens have variable expression patterns and not a single neoantigen is known to present in all MSI cells. Multivalent vaccine development against the immunogenic FSP neoantigens showed an effective immunopreventive strategy for MMR deficient cancers in preclinical mouse model [12]. Children who carry biallelic mutations in MMR genes develop CMMRD characterized by early development of pediatric lymphomas, brain tumors, and intestinal cancers [46]. Accumulation of “ultra-hypermutation” in those pediatric cancers present a compelling scenario for FSP neoantigen based prevention of cancers for CMMRD patients [47].

Germline mutation carriers of *BRCA1/2* show defect in homologous recombination (HR) repair and have an increased risk of developing breast, ovarian, pancreatic, or prostate cancers. Unique combinations of mutation types termed as “mutational signatures” arise due to different mutational processes. Currently, six sets of signatures (single base substitution, double base substitution, small insertion and deletion, copy number, structural variation and RNA single base substitution) are catalogued at COSMIC (Catalogue of Somatic mutations in Cancer, https://cancer.sanger.ac.uk/cosmic/). Signatures of somatic mutations have been identified in HR deficient *BRCA1/2*-associated breast cancer and *BRCA2*-mutated prostate cancers [48, 49]. Single base substitution (SBS) mutations of signature 3, accompanied by deletions of > 3 bp with microhomology at rearrangement breakpoints, and SBS signature 8 together with CC>AA double nucleotide substitutions are associated with the absence of BRCA1 and BRCA2 functions [48]. These somatic mutation signatures may serve as neoantigens for generating vaccines that may target BRCA1/2 deficient cancers. Analysis of mutation signatures among *BRCA1*-mutated breast cancers identified *PIK3CA* H1047R, E545K, E542K, and N345K mutations as the most recurrent among *BRCA1*-negative groups and *TP53* R175H is the most frequent mutation for germline *BRCA1*-mutated breast cancer [50]. These candidate neoantigens may be used to develop a preventive cancer vaccine in *BRCA1*-mutated carriers after validating their antigenicity and assessment of their ability to regulate cancer progression.

Besides the tumors arising in germline carriers of certain cancer predisposing genes, there are certain cancers that share driver mutations of certain cancer-causing genes. One of the most common driver mutations is *KRAS*^*G12D*^ that drives over half of pancreatic cancers and certain percentages of colon and lung cancers [51]. A phase I clinical trial has been initiated recently to study the safety and immune response using mutant KRAS peptide to the patients who have familial history of developing cancers or carrier of *BRCA2*, *ATM* or *PALB2* mutation (NCT05013216).

In the future, targeting of individuals with other hereditary cancer syndromes (e.g., Li-Fraumeni syndrome, neurofibromatosis etc.) will be an important area of focus for cancer immunoprevention [52, 53]. Besides that, individuals with precancerous lesions or with exposure to environmental or occupational carcinogens (e.g., Barrett’s esophagus, intraductal papillary mucinous neoplasms, monoclonal gammopathy of undetermined significance (MGUS), smokers, or peoples with high exposure to air pollution) constitute additional high-risk populations who could potentially benefit from immunoprevention. As next generation sequencing (NGS) data is continuing to expand the spectrum of cancers linked to various germ-line mutations and other sporadic mutations, it may be possible to identify more common immunogenic neoantigens that can be used for immunoprevention studies of mutation carriers.

Further, genetic alterations during cancer development can also affect the outcome of the immunopreventive agents. In a preclinical mouse model of carcinogen induced oral cancer, the mutational status of *Tp53* dictates the immunopreventive effect of anti-PD1 antibodies [54]. This suggests the importance of genetic profiling and personalized cancer immunoprevention in the future.

## Preclinical studies of cancer immunoprevention

Standard preclinical experiments investigate how immunological maneuvers delay or lower tumor incidence in young, tumor free mice that are susceptible to development of tumors. Mouse models that are used for cancer immunoprevention studies are mainly of two types: conventional carcinogen induced cancer models [55, 56] and genetically engineered cancer prone models [9, 12, 57, 58]. To elicit immune response against tumor cells, various approaches have been successful in murine models, like administration of monoclonal antibodies against pro-tumorigenic immune components [55, 56, 58] e.g.; blockade of immune checkpoint programmed death receptor 1(PD-1) inhibited the progression of oral premalignant lesions to oral cancers in murine oral carcinogenesis models [56, 59, 60]. Providing active stimuli or vaccine approach using tumor specific antigens [9, 10, 12, 61–63] is another way to achieve immunoprevention. Vaccine based approaches have used different technologies: providing cell extract, DNA, protein, peptides or fusion cells of tumor and dendritic cells (DC). Different vaccine-based approaches elicited both humoral and cytotoxic T lymphocyte (CTL) responses and provided positive outcomes in prevention studies. A summary of selected preclinical cancer prevention studies using different approaches is listed in Table 2 and appendix 1.

**Table 2:** Some preclinical studies of cancer prevention using different immunological approaches

Immunological approaches	Mouse models	Type of cancer	Outcome	References
Immune modulation				
Anti-PD1 antibody	4-nitroquinoline-1 oxide (4NQO) induced model	Oral cancer	Activate the immune system, reduces early precancerous and invasive cancer oral lesions	[59]
IL-12	Transgenic mice expressing rat HER-2/*neu*	Breast cancer	Delayed tumor onset and reduced tumor multiplicity	[133]
Vaccine				
Peptide vaccine against Epidermal growth factor receptor (EGFR)	Mouse model with inducible expression of full-length human EGFR with the L858R mutation	Non small cell lung cancer	Delays tumor development, elicits strong, specific immune responses	[11]
DNA vaccine targeting CYP26a1	4T1 syngeneic mouse model	Breast carcinoma	Inhibits tumor growth and progression, both humoral and cellular immune responses are elicited	[134]
Combination of vaccine and immune modulation				
Tumor cell vaccination and IL-12 treatment	HER-2/neu transgenic mice	Breast cancer	Inhibited tumor onset and progression to malignant mammary carcinoma	[135]
Protein-based vaccine targeting Ascl2 and anti-PD-1	*Apc*^+*/Min−FCCC*^ mice	Colorectal cancer	Decreased the formation of spontaneous colon adenomas	[9]

Lynch syndrome (LS) patients have a high mutation rate due to mutations in one of the DNA mismatch repair (MMR) genes resulting in microsatellite instability–induced (MSI-induced) shifts of the translational reading frame and giving rise to numerous neoepitopes. In a LS mouse model, vaccination against multiple neoepitopes generated due to defective mismatch repair elicited effective immune response, delayed intestinal polyp formation and increased survival [12]. Tumors from vaccinated mice showed elevated levels of CD4 positive cells as well as higher CD8 T cell counts, supporting the effective induction of adaptive immunity [12]. This study showed the development of memory T-cell response indicating the possibility of developing a common vaccine for MSI-high population. Another example of possible development of a common vaccine is the use of a multivalent peptide vaccine that can target multiple cancer causing mutations. Mutations of the Kirsten rat sarcoma viral oncogene homolog (KRAS) are most frequently observed among lung cancer patients with smoking history [64]. A vaccine targeting multiple epitopes of the KRAS molecule in a mouse model of a KRAS-driven lung tumor elicited Th1 immune response and reduced tumor burden and tumor number by > 80% in a genetically engineered mouse model for *KRAS* driven lung adenocarcinoma [57]. This vaccine may in the future be useful against many other KRAS-driven cancer types.

For more effective immunopreventive approaches, the combination of vaccine with other immunological approaches has been investigated in preclinical mouse models. The *Apc*^+*/Min−FCCC*^ mouse model is susceptible to spontaneous colon tumorigenesis and vaccine administration against achaete-scute family bHLH transcription factor 2 (Ascl2), an early colon cancer antigen elicited strong humoral and cellular immune responses. Vaccine in combination with immune checkpoint inhibitor anti-PD1 decreased colon adenoma formation and increased the infiltration of CD3+ T lymphocytes in colon adenomas [9]. While combinatorial approaches can lead to advanced clinical benefit, the timing of administration can dictate the outcome. Combining an OX40 agonistic antibody (aOX40) with engineered IL12-producing allogeneic HER2/neu-positive cell vaccine affected immunopreventive efficacy in HER2/neu transgenic BALB-neuT mice depending on the schedule of administration of aOX40. Administration of aOX40 after cell vaccination significantly enhanced immunoprevention of HER2/neu-driven mammary carcinogenesis whereas concomitant administration impairs the vaccine efficacy [65]. This suggests the thorough interrogation of preclinical models before planning clinical trials of combined approaches.

Various preclinical studies demonstrate that vaccination using TAA or TSA can elicit strong and specific immune responses with promise of immunoprevention in individuals with elevated risk of developing certain cancers. However, many preclinical immunoprevention studies of non-viral cancers using vaccine-based approaches are directed against transgenic products rather than endogenous molecules, minimizing the chance of autoimmunity. Early clinical trials for those immunoprevention studies will thus require special attention to monitor the risks of triggering autoimmunity.

## Clinical studies of immune based cancer prevention

Data from clinical studies of immunoprevention of non-viral mediated cancer is very limited. However, some proof-of-principle immunoprevention clinical studies have been carried out with promising results (Table 3 and appendix 2). We have also discussed here some clinical studies that are focused on prevention of recurrence due to their potential future use for immunoprevention. Early phase studies using a single antigen such as MUC1 or HER2 in colorectal adenoma patients with resected polyps or in DCIS respectively, showed safety and induction of antigen specific immune responses (NCT00773097) [26, 66]. Multiple phase I clinical studies using single antigens like MUC1 peptide vaccine in preventing lung cancer in current and former smokers (NCT03300817), EGF-based vaccine on the prevention of lung cancer development in high-risk patients, or recurrence in patients with stage IB to IIIA NSCLC (NCT04298606), alpha-lactalbumin (aLA) based vaccine in patients with early stage triple negative breast cancers (TNBC) (NCT04674306) are currently ongoing (appendix 2).

**Table 3:** Completed clinical trials for prevention of non-viral cancer occurrence, progression or remission

Targeted cancers	Antigens	Type of vaccine	Type of study	Participants	ClinicalTrials.gov identifier	Results
Breast Cancer with low to intermediate HER2 expression	HER2	Peptide	Phase III	People with HER2 negative breast invasive adenocarcinoma with no sign of disease	NCT01479244	Completed. No significant difference in disease free survival between vaccinated and placebo group [69]
Colorectal adenoma	MUC1	Peptide	Phase II	Patients with colorectal adenoma at the age of 40–70 years	NCT00773097	Completed. About 43.6% patients showed vaccine elicited MUC1 specific immune response [26]
Breast cancer	HER2	Peptide	Phase I	Node negative breast cancer patient without evidence of disease	NCT00854789	Safe and have clinical efficacy [70]
Prostate cancer	Prostate-specific antigen (PSA) and a triad of human T-cell costimulatory molecules (B7.1, ICAM-1, and LFA-3, designated TRICOM)	Viral	Phase II	Prostate cancer patients with stage < T2a	NCT02326805	Well-tolerated but did not elicit significant immune responses [71]
Melanoma	Tyrosinase, gp100 antigen, and MART-1 antigen	Peptide	Phase III	Patients with resected high-risk melanoma	NCT01989572	No significant improvement of recurrence free survival or overall survival[68]
Melanoma	gp100 and MAGE-3	Peptide	Phase II	Patients who are clinically rendered free of disease after surgery of stage IIb-IV melanomas	NCT00254397	Completed
Breast and ovarian cancer	Folate binding protein E39 and J65	Peptide	Phase I	Patients who have been treated and are without evidence of disease	NCT02019524	Safe, elicits immune response[73]
Melanoma	Personalized neoantigen	Peptide	Phase I	Patients after complete surgical resection of lesions	NCT01970358	Induce long term T cell response and tumor specific cytotoxicity [136]

Several clinical studies using multiple epitopes to induce immune response have shown the safety and ability to induce immune responses (NCT02780401) [67]. A phase I trial using DNA plasmid-based vaccine (WOKVAC) encoding epitopes from breast cancer antigens IGFBP-2, HER2, and IGF-1R that are expressed in pre-invasive and high-risk breast lesions showed that it can elicit significant immune responses in stage II/III breast cancer patients (NCT02780401) and has promise of preventing cancer development in high-risk patients. A Phase II study of this vaccine in combination with chemotherapy and HER2-targeted monoclonal antibody therapy to treat patients with HER2 positive breast cancer is ongoing (NCT04329065). Another phase I clinical study using a pooled mutant-KRAS peptide vaccine is ongoing in high-risk group for developing pancreatic cancer (familial pancreatic cancer relative or germline mutation carrier for pancreatic cancer susceptibility genes like *BRCA2*, *p16/CDKN2A*, *ATM*, *PALB2*) to evaluate safety and immunogenicity of the vaccine (NCT05013216). A phase IIb clinical trial for preventing cancer in individuals with Lynch syndrome is ongoing (NCT05419011). This trial is using multiple antigens that are expressed in precancer and cancer cells (CEA/MUC1/Brachyury) along with IL-15 Superagonist N-803 to measure the incidence of adenomas and elicited immune responses. Multiple epitopes associated with breast cancer stem cells in a DNA vaccine is now being used in a phase II trial to test their immunogenicity in triple negative breast cancer patients with high risk of recurrence (NCT05455658).

Vaccination using neoantigens that are tumor specific has been a recent focus of immunoprevention in high-risk groups. As described above, Lynch syndrome patients inherit mutations in mismatch repair genes and have higher risks of developing colorectal and certain types of other cancers. Preclinical studies using multiple neoantigens have showed effective immune response and reduction in tumor burden in a mouse model deficient in mismatch repair [12]. A phase Ib/II clinical trial using Nous-209, a viral vector-based vaccine encoding frameshift peptides (FSPs) has been initiated recently to evaluate safety and neoantigen-specific immunogenicity in Lynch syndrome patients (NCT05078866).

However, some interventional clinical studies using vaccines have failed to prevent recurrence of disease. A phase III trial with a multipeptide vaccine against tyrosinase, gp100 antigen, and MART-1 antigen in patients with resected melanoma showed that this vaccine was ineffective either alone or in combination with immunomodulatory molecule like GM-CSF in preventing recurrence of disease [68]. Another peptide vaccine against HER-2 showed clinical efficacy in phase I/II trial (NCT00854789), however failed to increase recurrence free survival of breast cancer patients in a phase III trial (NCT01479244) [69, 70]. In a phase II trial with a vaccinia viral vector-based vaccine that contains prostate specific antigen (PSA) and three T-cell costimulatory molecules, vaccine was well tolerated but failed to elicit significant T-cell responses compared to placebo in patients with localized prostate cancer (NCT02326805) [71]. The immune status of the patients or the lack of immune response to antigens may play role in limited efficacy of these vaccines in therapeutic settings.

Thymic atrophy can be another potential cause of low clinical efficacy of the vaccines. It has been shown that ablation of sex hormones by a LHRH (luteinizing hormone-releasing hormone) agonist treatment can increase thymus function and thus enhance the rate of T cell regeneration [72]. A phase II clinical trial has been recently completed to evaluate the immune responses of melanoma-specific peptide vaccines, gp100 and MAGE-3 in the presence or absence of Leuprolide, a LHRH-agonist (NCT00254397) in patients with stage II-IV melanoma whose lesions are surgically removed.

An approach to overcome immune tolerance is to use highly immunogenic antigens. However, repeated stimulation with highly immunogenic peptides may cause T-cell exhaustion. A phase Ib clinical trial using an attenuated version of an immunogenic peptide of Folate binding protein (FBP) in combination with wild-type peptide in disease-free breast and ovarian cancer patients after standard therapies elicited better immune response compared to wild-type peptide alone (NCT02019524) [73]. This showed the promise of this strategy, but leaves questions remaining regarding dosing and sequencing of the attenuated peptide vaccine.

Personalized neoantigen vaccines have been the focus of recent research. A phase I study among melanoma patients using personalized neoantigens demonstrated long term T cell response and effective cancer control [68]. Theoretically, cancer vaccine trials are suitable for patients with an intact immune system and disease detected at early stage. Patients who have developed invasive cancers may have compromised immune systems making it difficult to show effectiveness of vaccines. However, it is challenging to conduct trials for these vaccines among healthy population. In the future, more clinical trials with full consideration of the patient’s immune system function, immune health and tumor burden are anticipated to develop a potent strategy for cancer immunoprevention in highest risk populations.

## Other considerations for cancer immunoprevention

### Vaccine design platform

Different platforms have been used for cancer prophylactic vaccine development but data from head-to-head trials for the selection of the ideal vaccine platform is limited. Broadly, five categories of vaccines based on how they work to trigger immune responses have been used: cell-based, viral-vectors, peptide, DNA and mRNA [74]. Each category has own advantages and disadvantages and are summarized elsewhere [75]. The most commonly used platform is the peptide-based vaccine platform. However, the success of peptide vaccine relies on the choice of correct peptide and the best adjuvant to initiate immune responses. DNA vaccines are simple to design, more stable and soluble and can be designed to act as both an antigen and adjuvant. The RNA vaccine platform is comparatively safer and easier to deliver in cells, because it does not integrate into host genome and does not need to be delivered into the nucleus as compared to the DNA platform. Moreover, it is easier to manufacture, can encode multiple epitopes and has a built-in adjuvant function through TLR7 and TLR8 stimulation [76]. After the successful use of mRNA based vaccines during COVID-19 pandemic, and considering rapid production, scalability and ability to elicit both CD4^+^ and CD8^+^ T cell responses, mRNA based vaccine approaches for cancer immunoprevention has become an active area of investigation [75].

### Delivery of antigens

Several delivery systems have been tried in developing cancer vaccines to deliver antigens in the cells. A variety of nanoparticle-based systems like liposomes, micelles, carbon nanotubes, polymeric nanoparticles have been explored as drug delivery platforms [77]. Liposomes are popular vaccine delivery systems and are used to deliver DNA, RNA and antigens. Liposomes with different properties can be constructed by changing the lipid composition, charge, size and surface properties [78]. BioNTech has developed a lipid-based nanoparticle formulation for efficient targeting of RNA into dendritic cells that are resistant to degradation at 37 °C [79]. Another delivery system to deliver antigens into cells is found in self-assembling peptides that can spontaneously form ordered structures in response to changes in temperature, pH, ionic strength, solvent and co-assembling molecules [80]. Engineered microbial systems has recently been used to deliver array of tumour-specific neoantigen derived epitopes to induce anti-tumor immunity and inhibit immunosuppressive mechanisms [81].

### Immunomodulatory agents and immunoadjuvants

During antigen delivery another consideration is co-administration of immunomodulatory agents or prevention agents to increase the efficacy of vaccine. Combinatorial application of cytokines/chemokines (GM-CSF, IL12, IL15) or immunostimulatory molecules (CCR7, CD70, CD137, etc.) along with vaccine targets can improve the efficacy of vaccines [82–84]. Immunomodulator CA170 is a synthetic tripeptide antagonist of V-domain Ig Suppressor of T cell Activation (VISTA). VISTA controls T-cell activation to limit overstimulation of immune system after antigen exposure using a pathway distinct from PD1/PD-L1 pathways. Molecules like CA170 that disrupt VISTA signaling promote anti-tumor immunity [85]. Lung cancer development was almost completely suppressed when CA170 was used in combination with a KRAS peptide vaccine in a carcinogen induced lung cancer mouse model [86].

In a preclinical Lynch syndrome study, peptide vaccines against neoantigens along with chemopreventive agent aspirin or naproxen increased survival of mice compared to individual agent treatment [12]. Tumor cells heavily rely on glutamine metabolism and blocking glutamine metabolism improves anticancer immunity. In a preclinical study using syngeneic and GEM models, it was shown that the combination of Epidermal Growth Factor Receptor (EGFR)-peptide vaccine and an antagonist of glutamine utilizing enzymes is more effective in decreasing tumor burden and increasing anti-tumor immune responses compared to either agents alone [87]. Inhibition of cholesterol mechanism enhances the effector function of CD8+ T cells [88]. Combination of multipeptide KRAS vaccine and cholesterol metabolism inhibition enhanced the efficacy of vaccine in KRAS-driven lung cancer mouse models [89].

The success of immunopreventive vaccines require formulations that are effective in generating effective immune responses or boosting the existing responses along with their ability to overcome immune evasion mechanisms. Adjuvants are needed to attract immune cells to the site of injection and trigger the activation of APCs. The discovery and development of novel adjuvants with sufficient immunomodulatory activities and without adverse toxicities are of immense importance for cancer immunoprevention, considering the high toxicity bar when administering to high-risk individuals who are relatively healthy compared to cancer patients.

Toll-like receptor (TLR) agonists have been widely explored as adjuvants. TLRs are expressed in innate cells and act as pattern recognition receptors (PRRs) to induce immune responses when they recognize pathogen associated molecular pattern molecules (PAMPs). TLR agonists mimic microbial stimulation of immune responses and have shown to increase the magnitude of CD8+ T-cell responses [90]. Many TLR agonists are in trial as adjuvants for cancer vaccines, like the TLR3 agonist: polyinosinic–polycytidylic acid with polylysine and carboxymethylcellulose (Poly-ICLC), TLR4 agonist: monophosphoryl lipid A (MPLA), TLR7 agonist: imiquimod, TLR7 and TLR8 agonist: resiquimod, and TLR9 agonist: CpG oligodeoxynucleotide (CpG) [76, 91, 92]. In a preclinical mouse model, a TLR3 agonist, Poly-ICLC was used in combination with CD40 agonist to amplify DC vaccinations and boost T-cell immune responses [93].

The 4-1BB (CD137) receptor ligand (4-1BBL) is a trimeric transmembrane protein expressed in antigen presenting cells and upon binding to its receptor in T-cells delivers a robust costimulatory signal. A recombinant chimeric protein (SA-4-1BBL) containing the extracellular domains of murine 4-1BBL fused to a modified form of core streptavidin has generated robust CD8^+^ T effector and memory responses in several preclinical tumor models as an adjuvant with tumor associated antigen vaccines [94–97]. The chimeric protein SA-4–1BBL alone also protected mice against tumor challenge in various preclinical mouse models via non-specific activation of CD4^+^ T and natural killer cells demonstrating its ability to prime the immune system for cancer prevention [98].

A number of immunostimulatory cytokines like IL-2, IFN, IL-12, IL-15, and granulocyte–macrophage colony stimulating factor (GM-CSF) have been investigated as potential adjuvants. Among these, GM-CSF is the most studied and has been included in many trials [99–103]. In preclinical studies GM-CSF was found to be involved in enhancing adaptive immune response by recruiting DCs to the injection site and promoting the maturation of DCs and antigen presentation [104].

A major challenge for selecting the adjuvant that would be best suited for a specific vaccine is the lack of complete information about the immune profiles of adjuvants. To address these challenges in adjuvant selection, in 2022, the National Institute of Allergy and Infectious Disease (NIAID) has initiated the Adjuvant Comparison and Characterization (ACC) program to establish a systematic approach for adjuvant immune profiling and rational selection of adjuvants for specific vaccines [105].

### Vaccine administration time

Administration of vaccine in an ideal window to elicit effective immune response against tumor cells is another challenge for cancer immunoprevention. Immune escape of tumor cells due to presence of immunosuppressive environment (T regulatory cells, tumor associated macrophages, myeloid-derived suppressor cells) is an important consideration to avoid resistance to cancer vaccines. Studies have shown that progression of precancerous lesion towards invasive cancer, e.g. progression of intraductal papillary mucinous neoplasias (IPMNs) to pancreatic ductal adenocarcinoma (PDAC) or progression of ductal in-situ carcinoma (DCIS) to invasive breast cancer is often associated with infiltration of immunosuppressive cells to tumor sites [17, 106–108]. Administration of vaccine at the earliest pre-malignancy stage to high-risk individuals or conducting initial safety and efficacy trials in patients with resected cancer can potentially overcome this challenge. Detection of early precancerous lesions in high-risk groups with advanced early detection methods can also make immunoprevention trials highly feasible [109].

## Conclusions and emerging opportunities

To address the burdens of cancer associated mortality, cancer immunoprevention seeks to use vaccine and other immunomodulatory agents to prevent initiation or intercept malignancy at early stage. In this review we discussed various considerations for immune based prevention approaches with evidence from preclinical and clinical studies. Cancer immunoprevention studies are still at early stages with very limited clinical study data. With the advancement of cancer genetics and further understanding of immune mechanisms, cancer vaccines for immunoprevention are likely to continue to emerge.

People with a high-risk for developing cancers such as those who have inherited *BRCA1/2* mutations or mutations in mismatch repair genes are natural choice for advancing the science of immunoprevention. In the near future, development of vaccines targeting multiple neoantigens or proposing combinatorial immunoprevention approaches may overcome the current challenges of immune evasion of tumors or central immune tolerance and improve clinical efficacy of vaccines. Neoantigens are often generated by passenger mutations and using multiple neoantigens to immunize will help to prevent immune evasion of tumors. Considering the complicated mechanisms of immune escape of cancers, targeting different stages of the cancer immunity cycle using combinatorial approaches (e.g., using drugs that will alter immunosuppressive tumor microenvironment along with neoantigen vaccine or boosting neoantigen presentation using immunomodulators like GM-CSF, anti-CD-40 etc.) may be more effective. A valuable source for tumor-specific antigens is transcripts initiated from epigenetically deregulated transposable elements (TEs) during cancer progression that results in a chimeric RNA product that joins TE sequence with gene sequence (TE-chimeric transcript). Identification of peptides translated from such TE-chimeric transcript may lead to strategies to target tumor-specific events [110, 111]. Identification of immunogenic non-canonical peptides, or Dark Matter [112], or cryptic peptides derived from non-canonical protein translations-especially from non-coding RNAs, intronic regions or physiologically untranslated regions (5’ UTRs, endogenous retroelements, pseudogenes etc.) in high risk groups may serve as targets for immunoprevention strategies. Alternately processed transcripts in precancer lesions can produce novel peptides downstream of frameshift mutations and can serve as “cryptic” neoantigens. The Cryptic peptides have not yet been extensively investigated, but it has been estimated that about 10–15% of all peptides presented by HLA-1 are cryptic peptides and are an excellent source of TSAs [113–115]. Further exploitation of these peptides may direct potential preventive approaches.

Immune checkpoint inhibition (ICI) has revolutionized the treatment of cancer especially for melanoma, renal and lung cancer [116]. ICI has not been widely investigated in prevention of cancer in high-risk patients. Progression of cirrhosis into hepatocellular carcinoma could be prevented in a mouse model with intermittent ICI intervention in one study [117]. ICI intervention has been associated with immune related adverse effects or toxicities. However, this may effectively be used either alone or in combination with vaccine in interception of early precancer lesions where immune systems are intact, once safe regimens are established [118].

In conclusion, much work remains to be done in defining the landscape for the development of a successful immunopreventive strategy of non-viral cancers. Multifaceted strategies including innovative approaches for stimulating effective and long-lasting immunity against tumors, strategic antigen selection, optimal selection of delivery system including nanoparticle-based delivery approaches and identifying suitable prime-boost approaches hold the potential to enhance cancer prevention. Identifying high-risk individuals as candidates for prevention or interception strategies is another challenge and work is ongoing for early detection and understanding the immune system in precancer environments. If successful, this immunoprevention strategy will have major implications in public health for millions worldwide.

## Supplementary Information

Supplementary Material 1.

## Data Availability

No datasets were generated or analysed during the current study.
