# Supplementary material for: Immunoprevention of non-viral cancers: challenges and strategies for early intervention
Source: Cancer Cell Int. 2025 May 28;25:196. doi: 10.1186/s12935-025-03817-8 (PMC12121240; doi:10.1186/s12935-025-03817-8)
Supplement: Supplementary file 1 — Supplementary Material 1. [file 12935_2025_3817_MOESM1_ESM.docx]

**Appendix 1. Preclinical studies for cancer immunoprevention**

| **Cancers targeted** | **Mouse Model** | **Agents** | **Conclusion** | **Reference** |
| --- | --- | --- | --- | --- |
| *K-Ras* mutant lung adenocarcinoma | K-ras^G12D^–mutant mice (CCSP^Cre^/LSL-K-ras^G12D^) CCSP: club cell secretory protein | anti–IL-1β antibody | Decreases tumor burden and tumor cell proliferation, antitumor phenotype of tumor microenvironment is elicited | [58] |
| Mismatch repair deficient colorectal cancer/ Lynch syndrome | Lynch syndrome mouse model Villin-Cre/Msh2^LoxP/LoxP^ | Four frameshift peptide neoantigens (Nacad [FSP-1], Maz [FSP-1], Senp6 [FSP-1], Xirp1 [FSP-1]) | Reduced tumor burden with increased adaptive immunity, prolonged overall survival. | [12] |
| Colorectal cancer | Apc^+/Min-FCCC^ model | Chimeric protein containing murine form of achaete-scute family bHLH transcription factor 2 (Ascl2) as antigen | Reduced colon adenocarcinoma formation and increase in CD3+ T lymphocytes | [9] |
| Oral squamous cell carcinoma (OSCC) | 4-nitroquinoline 1-oxide (4-NQO) murine model of oral carcinogenesis | Anti-CD40 monoclonal antibody | Reduce malignant transition of oral premalignant lesions (OPL), expansion of memory cytotoxic T lymphocytes and M1 macrophages | [55] |
| K-RAS driven lung adenocarcinoma | Inducible CCSP-TetO-*K-Ras^G12D^* mouse model. | Multipeptide vaccine targeting multiple epitopes of K-RAS. | Reduce lung tumorigenesis >80%, induce robust Th1 immune responses | [57] |
| Epidermal growth factor receptor (EGFR) mutations driven lung adenocarcinoma | Transgenic mouse model with inducible lung specific expression of EGFR mutations and syngeneic mouse model | Multiple peptides targeting different EGFR mutations (L858R, T790M, and Del19) | Reduced tumor burden, elicited Th1 responses and decreased suppressive Tregs | [137] |
| Epithelial ovarian carcinoma | TgMlSIIR-Tag (DR26) transgenic mice that expresses large T antigen (Tag) of SV40 under control of the AMHR2 promoter | Anti-Müllerian hormone receptor, type II (AMHR2-ED) | Inhibition of murine EOC growth and overall survival increase. Induction of AMHR2-ED–specific IgG antibodies | [63] |
| Oral squamous cell carcinoma (OSCC) | 4-nitroquinoline 1-oxide (4-NQO) murine model of oral carcinogenesis | Anti-PD1 monoclonal antibody | Prevents malignant progression of low-grade dysplastic lesions, increase in CD8^+^ and CD4^+^ T cells and CTLA-4^+^ T cells in their microenvironment | [56] |
| HER2-driven mammary carcinogenesis | FVB-huHER2-transgenic mice | DNA containing human/rat HER2 construct or whole cell vaccine using HER2 positive SK-OV-3 cell line | Both vaccines delayed tumor onset. Anti-huHER2 antibodies were induced by the DNA vaccine and the cell vaccine elicited interferon (IFN)-γ production. | [10] |
| Triple negative breast cancer (TNBC) | 4T1 syngeneic mouse model | Dendritic cell vaccine targeting α-lactalbumin | Inhibits tumor growth. | [138] |
| Triple negative breast cancer (TNBC) | C3(1)/Tag transgenic mouse model and syngeneic TNBC mouse model | Multiple peptides from Topoisomerase 2 alpha (TOP2A) | Reduced tumor incidences and slowed down tumor growth. Elicited cellular immune response and long-term memory response. | [139] |
| Mammary and salivary carcinoma | Mice *p53* knockout and *Her2/Neu* transgenic (*Trp53^-/-^; Tg Neu*) | Triplex DNA-based vaccine (Tri-DNA), consisting of the combination of three gene components (a transmembrane-extracellular domain fragment of the Neu gene, IL-12 genes, and the H-2D(q) allogeneic MHC gene) | Prevention of salivary and mammary tumor development, helper T cell type 1 (Th1)-polarized response | [62] |
| Breast carcinoma | Allograft and intravenous metastasis model | Overlapping synthetic peptides for tumor protein D52 (TPD52) | Prolonged survival of vaccinated mice and induces CTL responses with anti-tumor activity | [140] |
| Breast carcinoma | BALB-neuT mice overexpressing rat Her2/neu oncogene | Allogeneic Her2/Neu expressing tumor cells engineered to release interleukin 12 (IL12) | >80% of tumor free 1-year-old mice free, high IFN-γ and IL-4 and a strong anti-HER-2/neu antibody response | [61] |
| Basal cell carcinoma | Ptch1*^+/-^* | Hedgehog-interacting protein (Hip1) recombinant polypeptide | Reduces number of basal cell carcinomas, induce B and T-cell responses. | [141] |
| Tumors induced by hepatocellular carcinoma cells | Allograft in BALB/c mice | Fusion of hepatocellular carcinoma (H22) cells and dendritic cells (DC) | Inhibit tumor growth, induce CTL response | [142] |

**Appendix 2. Ongoing clinical trials for prevention of non-viral cancer occurrence, progression or remission**

| **Targeted Cancers** | **Antigens** | **Type of vaccine** | **Type of Study** | **Participants** | **ClinicalTrials.gov identifier** |
| --- | --- | --- | --- | --- | --- |
| Lynch syndrome (colorectal, endometrial, other cancers) | 209 frameshift antigens | Viral | Phase Ib/II | People with clinical diagnosis of Lynch syndrome or “Lynch-like syndrome” with no evidence of active or recurrent cancer | NCT05078866 |
| Pancreas | KRAS | Peptide | Phase I | People in high-risk group who have either mutation or familial history of pancreatic cancer | NCT05013216 |
| Triple-negative breast cancer | 𝛂-lactalbumin | Protein | Phase I | People with triple-negative breast cancer with high risk of recurrence | NCT04674306 |
| Non-small cell lung cancer | Epidermal growth factor (EGF) | Protein | Phase I | High risk group of lung cancer with no active or recurrent cancer | NCT04298606 |
| Lung Cancer | MUC1 | Peptide | Phase I | High risk group of lung cancer (current and formal smokers) | NCT03300817 |
| Triple negative breast cancer | CD105, Yb-1, SOX2, CDH3, MDM2 polyepitope | DNA | Phase II | People with triple-negative breast cancer with high risk of recurrence | NCT05455658 |
| Node positive, HER2 negative breast cancer | IGFBP, HER2, IGF1R | DNA | Phase I | People with node positive, non- metastatic, HER2 negative breast cancer with no sign of disease and risk of recurrence | NCT02780401 |
| Bladder cancer | DEP domain containing 1(DEPDC1) and M phase phosphoprotein 1(MPHOSPH1) | Peptide | Phase II | Patients with low grade bladder tumor with DEPDC1 and MPHOSPH1 expression | NCT00633204 |
| Lynch syndrome | CEA, MUC1, Brachyury | Adenoviral | Phase II | People with clinical diagnosis of Lynch syndrome or “Lynch-like syndrome” with no evidence of active cancer for 6 months | NCT05419011 |
| Hepatocellular carcinoma | Personalized neoantigen | mRNA | Phase I | Patients with hepatocellular carcinoma IIb/IIIa and lesion have been removed by surgery | NCT05761717 |
